# Effects of Ingestion of Different Amounts of Carbohydrate after Endurance Exercise on Circulating Cytokines and Markers of Neutrophil Activation

**DOI:** 10.3390/antiox7040051

**Published:** 2018-04-02

**Authors:** Kumpei Tanisawa, Katsuhiko Suzuki, Sihui Ma, Saki Kondo, Susumu Okugawa, Mitsuru Higuchi

**Affiliations:** 1 Department of Physical Activity Research, National Institutes of Biomedical Innovation, Health and Nutrition, Tokyo 162-8636, Japan; tanisawa@nibiohn.go.jp; 2 Research Fellow of Japan Society for the Promotion of Science, Tokyo 102-0083, Japan; 3 Faculty of Sport Sciences, Waseda University, Tokorozawa 359-1192, Japan; mhiguchi@waseda.jp; 4 Institute of Advanced Active Aging Research, Tokorozawa 359-1192, Japan; 5 Graduate School of Sport Sciences, Waseda University, Tokorozawa 359-1192, Japan; masihui@toki.waseda.jp; 6 Department of Life Sciences, Graduate School of Arts and Sciences, The University of Tokyo, Tokyo 153-8902, Japan; saki-kondo@g.ecc.u-tokyo.ac.jp; 7 Products Research & Development Laboratory, Asahi Soft Drinks Co., Ltd., Moriya 302-0106, Japan; susumu.okugawa@asahiinryo.co.jp

**Keywords:** exhaustion, leukocyte, inflammation, muscle damage

## Abstract

We aimed to examine the effects of ingestion of different amounts of carbohydrate (CHO) after endurance exercise on neutrophil count, circulating cytokine levels, and the markers of neutrophil activation and muscle damage. Nine participants completed three separate experimental trials consisting of 1 h of cycling exercise at 70% $\overset{\cdot }{\text{V}}$O_2_ max, followed by ingestion of 1.2 g CHO·kg body mass^−1^·h^−1^ (HCHO trial), 0.2 g CHO·kg body mass^−1^·h^−1^ (LCHO trial), or placebo (PLA trial) during the 2 h recovery phase in random order. Circulating glucose, insulin, and cytokine levels, blood cell counts, and the markers of neutrophil activation and muscle damage were measured. The concentrations of plasma glucose and serum insulin at 1 h after exercise were higher in the HCHO trial than in the LCHO and PLA trials. Although there were significant main effects of time on several variables, including neutrophil count, cytokine levels, and the markers of neutrophil activation and muscle damage, significant time × trial interactions were not observed for any variables. These results suggest that CHO ingestion after endurance exercise does not enhance exercise-induced increase in circulating neutrophil and cytokine levels and markers of neutrophil activation and muscle damage, regardless of the amount of CHO ingested.

## 1. Introduction

Prevention of exercise-induced skeletal muscle damage is important for endurance athletes to regularly perform high-level training. Although mechanically induced muscle injury (e.g., overextension of sarcomeres and membrane damage) followed by local inflammation often occurs following eccentric contraction [1], non-mechanical (systemic) factors are suggested to be the main cause of muscle damage that is induced by prolonged endurance exercise [2]. One plausible mechanism for non-mechanical factor-induced muscle injury is the infiltration of inflammatory cells into skeletal muscle [3,4]. A number of experiments demonstrated that the infiltration of inflammatory cells, including neutrophils and macrophages, was observed in the injured muscle [5]; additionally, activated neutrophils and macrophages produce proinflammatory cytokines and reactive oxygen species [6,7,8], which induce inflammation and myofiber membrane lysis [9]. In humans, the exercise-induced increase in neutrophil count is highly correlated with an increase in circulating markers of muscle damage [2,10]. Recently, Kawanishi et al. have demonstrated that the depletion of neutrophils and macrophages attenuates muscle injury and inflammation after exhaustive exercise [3,4], highlighting that the exercise-induced infiltration of neutrophils and macrophages in skeletal muscle is a key factor in the development of muscle injury that is induced by endurance exercise. 

One strategy to attenuate endurance exercise-induced neutrophil mobilization, infiltration, and activation in skeletal muscle is carbohydrate (CHO) supplementation. Previous studies showed that CHO ingestion before/during prolonged exercise attenuated an exercise-induced increase in neutrophil count [11,12,13,14,15]; interleukin-6 (IL-6), which is a potent mobilizer of neutrophils [2,11,16,17]; chemokines [2,11,18]; neutrophil activation [2,13,19]; and, muscle damage [2,15,20]. These findings suggest that CHO ingestion before/during exercise is useful to prevent not only a decline in endurance exercise performance by sparing muscle glycogen stores, but also exercise-induced muscle damage. However, several studies demonstrated that CHO ingestion after eccentric exercise enhanced the gene expression of chemokines, such as monocyte chemotactic protein-1 (MCP-1) and interleukin-8 (IL-8) in skeletal muscle [21], increase in circulating IL-6 [22] and interleukin-1β levels, and muscle soreness [23]. Although CHO ingestion after endurance exercise is recommended to enhance muscle glycogen synthesis [24,25], these findings suggest that post-exercise CHO ingestion may promote muscle injury via the promotion of neutrophil and macrophage infiltration in skeletal muscle. 

Whereas several studies reported that CHO ingestion after eccentric exercise enhanced the inflammation and muscle soreness, Costa et al. only investigated the effect of CHO ingestion after endurance exercise on neutrophil count and its activation [26,27]. They demonstrated that CHO ingestion after endurance exercise did not influence the exercise-induced increase in neutrophil count, but attenuated the decrease in bacterially stimulated neutrophil elastase release. However, it is still unknown whether CHO ingestion after endurance exercise increases various neutrophil-mobilizing cytokines (e.g., IL-6 and granulocyte colony-stimulating factor [G-CSF]) or chemotactic factors (e.g., MCP-1, IL-8, and complement component 5a [C5a]), and in vivo markers of neutrophil activation (e.g., myeloperoxidase and calprotectin). Furthermore, how different amounts of CHO after exercise enhance or suppress the mobilization and activation of neutrophils should be examined to determine the optimal amounts of CHO ingestion during the recovery from endurance exercise. 

The purpose of this study was to systematically examine the effects of different ingested amounts of CHO after endurance exercise on neutrophil count, circulating cytokine levels, and markers of neutrophil activation and muscle damage.

## 2. Materials and Methods

### 2.1. Experimental Design

We designed a double-blind randomized crossover placebo-controlled study. The participants were recruited to participate in the following three separate experimental trials: endurance exercise, followed by the ingestion of either (1) high-CHO beverage (HCHO trial); (2) low-CHO beverage (LCHO trial); or, a (3) placebo beverage (PLA trial). The experimental protocols were approved by the Ethics Committee of Waseda University and registered with Japanese University Hospital Medical Information Network number UMIN000025360. Written informed consent was obtained from all of the participants prior to their enrollment in the study.

### 2.2. Participants

Nine young men participated in this study. The participants were recreationally active and had no chronic diseases. The means (standard deviation) [range] of the participant characteristics were as follows: age, 22.4 (2.7) [21,22,23,24,25,26,27,28,29] years; height, 172.6 (6.5) [163.8–186.2] cm; body mass (BM), 64.5 (6.6) [55.1–79.0] kg; and maximal oxygen uptake ($\overset{\cdot }{\text{V}}$O_2_ max), 49.6 (6.3) [40.3–60.9] mL·kg^−1^·min^−1^. 

### 2.3. Preliminary Testing

At least one week prior to the experimental trials, $\overset{\cdot }{\text{V}}$O_2_ max was measured by a maximal graded exercise test using a cycle ergometer (Aerobike 75XLII; Combi, Tokyo, Japan). The graded cycling exercise began with a workload of 60–90 W, which was subsequently increased to 30 W/3 min, until exhaustion. During the incremental portion of the exercise test, expired gas was collected from participants. O_2_ and CO_2_ concentrations were measured and were averaged over 30-s intervals using an automated gas analyzer system (Aeromonitor AE-310S, Minato Medical Science, Tokyo, Japan). The highest value of $\overset{\cdot }{\text{V}}$O_2_ that was recorded during the exercise test was considered the $\overset{\cdot }{\text{V}}$O_2_ max (mL·kg^−1^·min^−1^), and the achievement of $\overset{\cdot }{\text{V}}$O_2_ max was accepted if at least three of the following four criteria were met: the $\overset{\cdot }{\text{V}}$O_2_ curve showed a plateau, despite increasing the work rate, the maximal heart rate was 95% of the age-predicted maximal heart rate (220—age [in years]), the respiratory exchange ratio was greater than 1.1, and the subject achieved a perceived exertion rating of 18 or greater.

### 2.4. Experimental Protocol

All of the participants completed three separate experimental trials; each trial was performed on a separate day at least a week apart. On the day of each trial, participants consumed a standardized breakfast (CHO, 74.9 g; protein, 16.2 g; fat, 26.3 g; total energy, 600 kcal) between 7:00 and 7:30 a.m. Participants arrived at the laboratory between 9:00 and 9:30 AM, and pre-exercise blood samples were drawn. After a warm-up for 3 min at 60–90 W, the participants exercised on a cycle ergometer for 60 min at a target intensity of 70% $\overset{\cdot }{\text{V}}$O_2_ max in a climate chamber (temperature, 22 °C; humidity, 50%). Participants ingested 100 mL of water every 15 min during exercise to avoid dehydration. Expired gas concentration was measured 10–15 min after the start of exercise to confirm the intensity of exercise. Heart rate and the ratings of perceived exertion (RPE) were also recorded every 15 min during exercise. After 60 min of exercise, participants rested in the sitting position for 2 h (exercise recovery) in the laboratory. During the recovery, participants consumed a 30% CHO (15% glucose + 15% maltodextrin) beverage (HCHO trial), 5% CHO (2.5% glucose + 2.5% maltodextrin) beverage (LCHO trial), or placebo beverage (PLA trial) at 15 min, 45 min, and 75 min after the end of exercise in each trial, which provided 1.2 g CHO·kg BM^−1^·h^−1^ (HCHO trial) and 0.2 g CHO·kg BM^−1^·h^−1^ (LCHO trial) during the 2 h recovery phase, respectively. The consumed amount of CHO in the HCHO trial is equivalent to the recommendation for CHO intake during early recovery from exercise [24], whereas the concentration of 5% CHO beverages in the LCHO trial was equivalent to that in commercially available sports drinks. The 5% CHO and the placebo beverages were sweetened with an artificial sweetener to emulate the taste of the 30% CHO beverage. All of the beverages were carbonated and packed in the same bottles in a blinded manner.

### 2.5. Blood Sampling and Analysis

Venous blood samples were collected by venipuncture before exercise (Pre), immediately after exercise (Post), and 0.5 h, 1 h, and 2 h after the end of exercise. Blood samples were collected into serum separation tubes or ethylenediaminetetraacetic acid (EDTA)-containing tubes. A portion of whole blood was used to analyze the hemoglobin and hematocrit levels and blood cell counts using an automatic blood cell counter (pocH100*i*, Sysmex; Kobe, Japan). The serum separation tubes were left to coagulate at room temperature for 30 min, whereas the EDTA-containing tubes for plasma separation were immediately centrifuged at 3000 rpm (approximately 1700× *g*) for 10 min at 4 °C. Serum and plasma were stored at −80 °C until analysis. The concentrations of plasma glucose, serum insulin, and serum myoglobin, and serum enzymatic activities of creatine kinase were determined by BML Inc. (Tokyo, Japan). Commercially available enzyme-linked immunosorbent assay (ELISA) kits were used to determine the plasma concentrations of cytokines and markers of neutrophil activation. IL-6 and G-CSF were analyzed using the Quantikine high-sensitivity ELISA kit (R&D Systems, Minneapolis, MN, USA). MCP-1 was analyzed using the Quantikine ELISA kit (R&D Systems, Minneapolis, MN, USA). IL-8 and C5a were analyzed using the OptEIA Human IL-8 and C5a ELISA Kit II (BD Biosciences, Franklin Lakes, NJ, USA). Myeloperoxidase, calprotectin, and elastase were analyzed using ELISA kits from Hycult Biotech (Uden, The Netherlands). Blood cell counts were adjusted according to percentage change in blood, whereas plasma and serum variables were adjusted according to the percentage change in plasma volume calculated from hemoglobin and hematocrit levels [28].

### 2.6. Statistical Analysis

The sample size was calculated using the program G*Power [29]. Nine subjects were required to detect an effect size of *f* = 0.5 for the within-between interaction, with a power of 0.8 and a significance level of 0.05 under the assumption of a correlation coefficient among repeated measures *r* = 0.7 and a nonsphericity correction of ε = 0.5. Other statistical analyses were performed with SPSS version 24.0 (SPSS Inc., Chicago, IL, USA). The Shapiro-Wilk test was performed to assess the normality of the data distribution, and non-normally distributed variables were log-transformed prior to analysis. $\overset{\cdot }{\text{V}}$O_2_ during each trial was compared using one-way repeated measures analysis of variance (ANOVA). Two-way repeated measures ANOVA was applied to determine the main effect of time and trial, and the interaction effect of time × trial on other variables. The Greenhouse-Geisser correction was used to adjust degrees of freedom when the assumption of sphericity was violated. A *post hoc* test with Bonferroni correction was used to identify the significant differences among mean values if a significant main effect or interaction was identified. Data are presented as mean (standard deviation), and statistical significance was set at *p* < 0.05.

## 3. Results

### 3.1. Physiological Variables

The $\overset{\cdot }{\text{V}}$O_2_ values during exercise in the HCHO, LCHO, and PLA trials were 33.9 (6.1) mL·kg^−1^·min^−1^ (68.4% $\overset{\cdot }{\text{V}}$O_2_ max), 33.9 (6.0) mL·kg^−1^·min^−1^ (68.3% $\overset{\cdot }{\text{V}}$O_2_ max), and 33.3 (5.8) mL·kg^−1^·min^−1^ (67.2% $\overset{\cdot }{\text{V}}$O_2_ max), respectively, with no significant differences among the three trials (*p* = 0.486). The heart rate and RPE steadily increased during exercise (main effect of time: *p* = 0.024 and *p* < 0.001, respectively), but there were no significant time × trial interactions for those variables (*p* = 0.612 and *p* = 0.761, respectively). The respective heart rates and RPEs at the end of exercise were 162 (22) beat·min^−1^ and 15.8 (2.0) for the HCHO trial, 166 (19) beat·min^−1^ and 16.2 (1.6) for the LCHO trial, and 166 (18) beat·min^−1^ and 16.0 (2.5) for the PLA trial.

### 3.2. Glucose and Insulin

**Figure 1:**
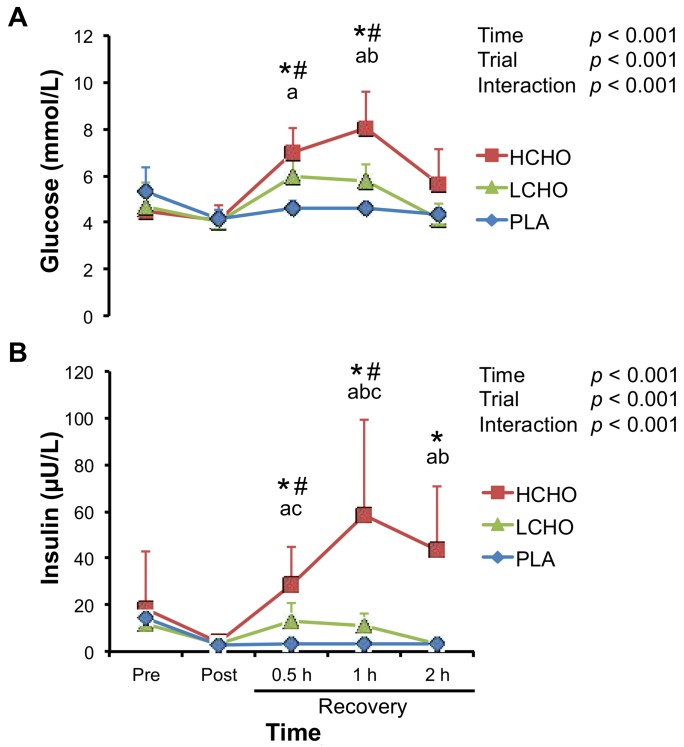
Changes in plasma glucose (**A**) and serum insulin (**B**) concentrations in response to endurance exercise. HCHO, high-carbohydrate trial; LCHO, low-carbohydrate trial; PLA, placebo trial; Pre, pre-exercise; Post, immediately after exercise; 0.5 h, 0.5 h after exercise; 1 h, 1 h after exercise; 2 h, 2 h after exercise. * *p* < 0.05 vs. Pre for HCHO trial. # *p* < 0.05 vs. Pre for LCHO trial. ^a^ *p* < 0.05, HCHO vs. PLA. ^b^ *p* < 0.05, HCHO vs. LCHO. ^c^ *p* < 0.05, LCHO vs. PLA.

Two-way repeated measures ANOVA demonstrated significant time × trial interactions for the plasma glucose and serum insulin concentrations (Figure 1). Plasma glucose concentrations at 0.5 h and 1 h after the end of exercise significantly increased when compared with post-exercise values in the HCHO and LCHO trials (Figure 1A). Plasma glucose concentrations at 0.5 h after the end of exercise were significantly higher in the HCHO trial than in the PLA trial, but not different between the LCHO and PLA trials. Furthermore, plasma glucose concentrations at 1 h after the end of exercise were higher in the HCHO trial than in the LCHO and PLA trials. Similarly, serum insulin concentrations at 0.5 h and 1 h after the end of exercise significantly increased when compared with post-exercise values in the HCHO and LCHO trials (Figure 1B). In the HCHO trial, serum insulin concentrations at 2 h after the end of exercise remained higher as compared to post-exercise values. Serum insulin concentrations at 0.5 h and 1 h after the end of exercise were significantly higher in the HCHO and LCHO trials than in the PLA trial. Furthermore, serum insulin concentrations at 1–2 h after the end of exercise were even significantly higher in the HCHO trial than in the LCHO trial.

### 3.3. Leukocytes

There were significant main effects of time on total leukocyte, neutrophil, and lymphocyte counts and neutrophil/lymphocyte ratio (Table 1). Peak concentrations were observed 1–2 h after the end of exercise for the total leukocyte and neutrophil count and neutrophil/lymphocyte ratio, whereas peak lymphocyte count was observed at post-exercise. However, these incremental patterns were similar among the three trials, and there were no significant time × trial interactions for these variables. No significant main effects of time on monocyte count were observed.

### 3.4. Neutrophil-Mobilizing Cytokines

**Figure 2:**
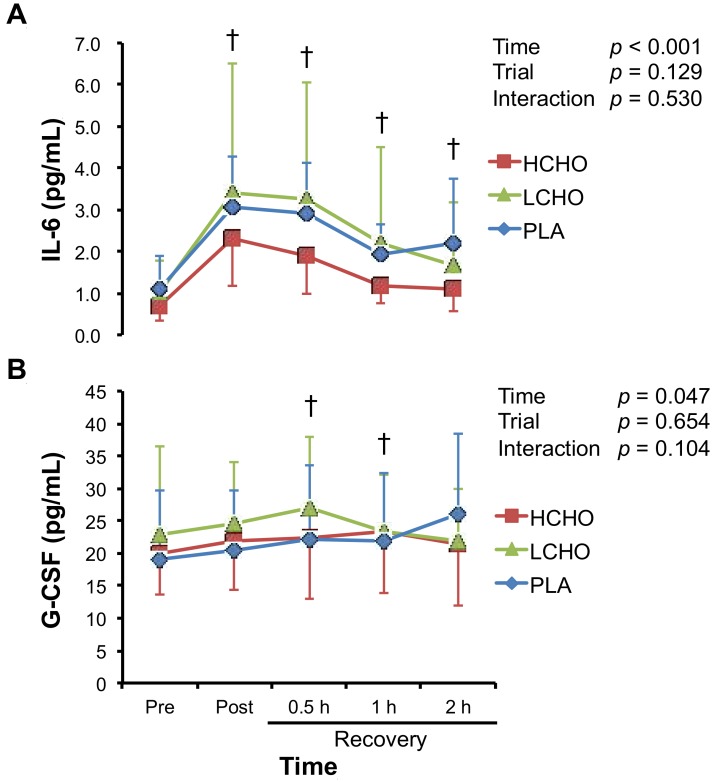
Changes in plasma interleukin-6 (IL-6) (**A**) and granulocyte colony-stimulating factor (G-CSF) (**B**) concentrations in response to endurance exercise. HCHO, high-carbohydrate trial; LCHO, low-carbohydrate trial; PLA, placebo trial; Pre, pre-exercise; Post, immediately after exercise; 0.5 h, 0.5 h after exercise; 1 h, 1 h after exercise; 2 h, 2 h after exercise. † *p* < 0.05 vs. Pre.

There were significant main effects of time on plasma IL-6 and G-CSF levels (Figure 2). The peak concentrations of IL-6 and G-CSF were observed at post-exercise and 0.5 h after the end of exercise. However, these incremental patterns were similar among the three trials, and there were no significant time × trial interactions for any of the variables.

### 3.5. Chemotactic Factors

**Figure 3:**
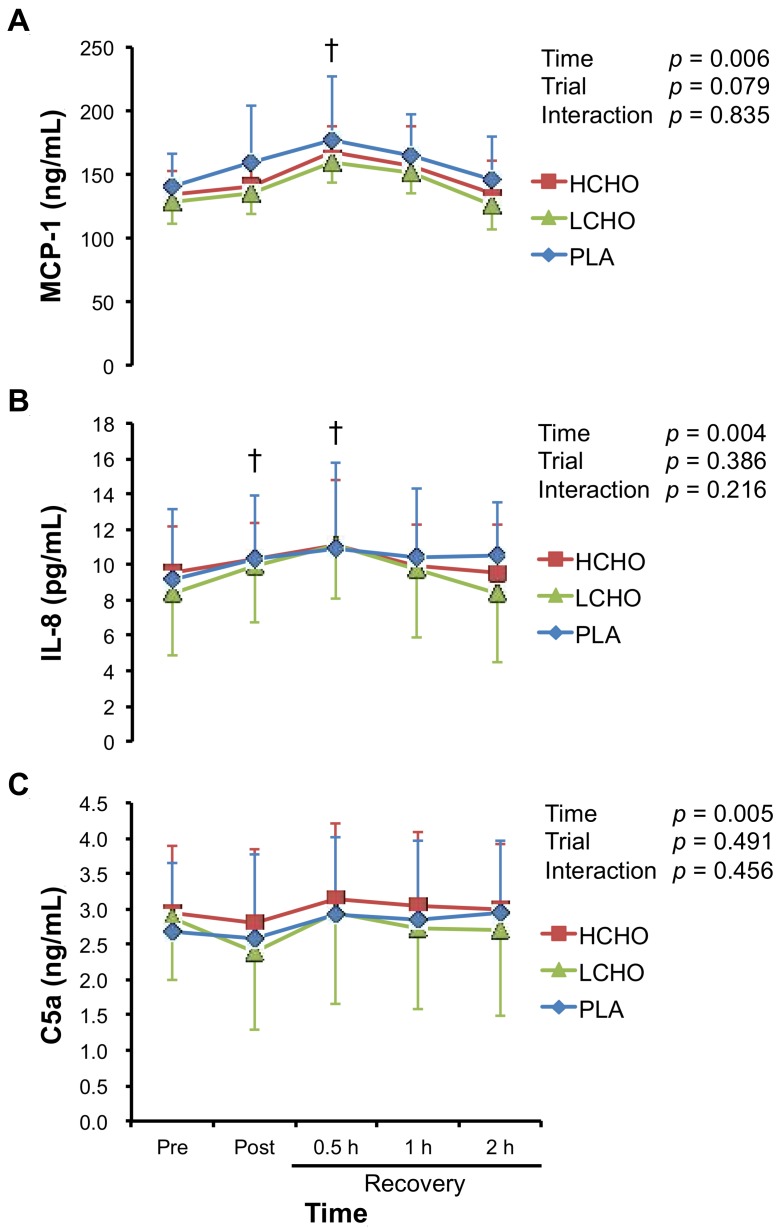
Changes in plasma monocyte chemotactic protein-1 (MCP-1) (**A**), interleukin-8 (IL-8) (**B**), and complement component 5a (C5a) (**C**) concentrations in response to endurance exercise. HCHO, high-carbohydrate trial; LCHO, low-carbohydrate trial; PLA, placebo trial; Pre, pre-exercise; Post, immediately after exercise; 0.5 h, 0.5 h after exercise; 1 h, 1 h after exercise; 2 h, 2 h after exercise. † *p* < 0.05 vs. Pre.

There were significant main effects of time on plasma MCP-1, IL-8, and C5a levels, for which concentrations peaked at 0.5 h after the end of exercise (Figure 3). However, these incremental patterns were similar among the three trials, and there were no significant time × trial interactions for any of the variables.

### 3.6. Markers of Neutrophil Activation

**Figure 4:**
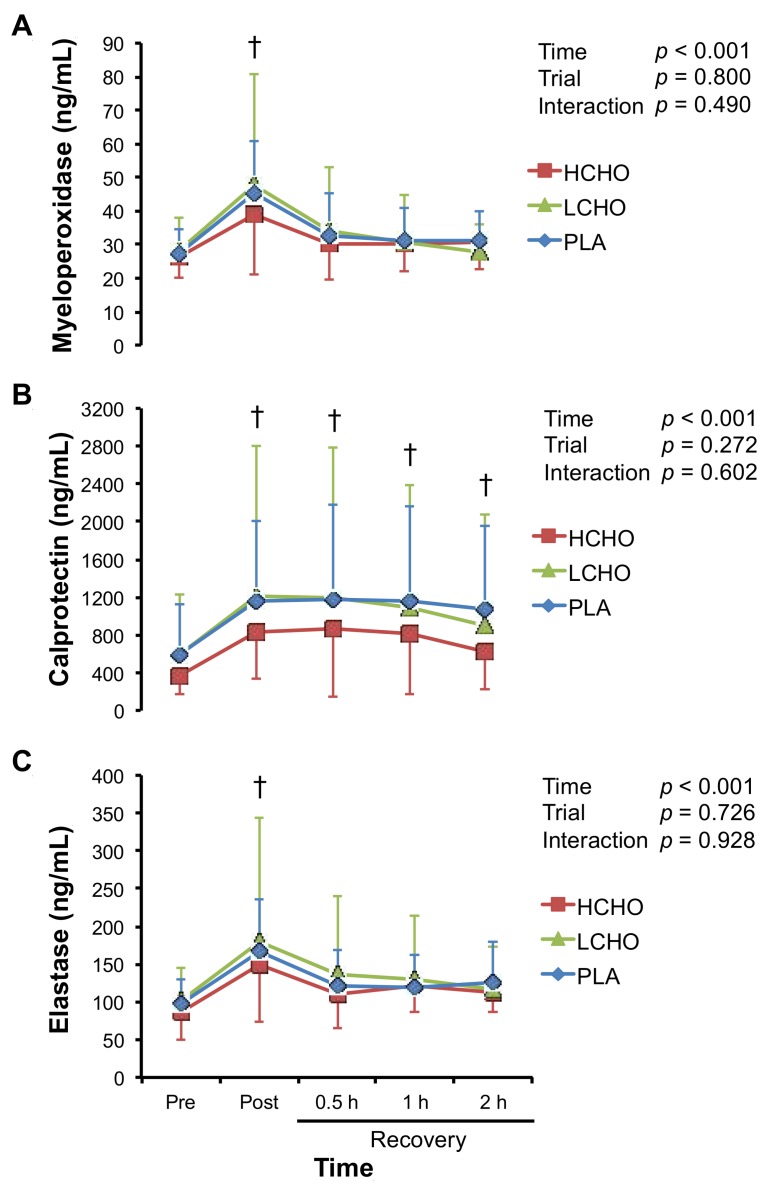
Changes in plasma myeloperoxidase (**A**), calprotectin (**B**), and elastase (**C**) concentrations in response to endurance exercise. HCHO, high-carbohydrate trial; LCHO, low-carbohydrate trial; PLA, placebo trial; Pre, pre-exercise; Post, immediately after exercise; 0.5 h, 0.5 h after exercise; 1 h, 1 h after exercise; 2 h, 2 h after exercise. † *p* < 0.05 vs. Pre.

There were significant main effects of time on plasma myeloperoxidase, calprotectin, and elastase concentrations, for which concentrations peaked at post-exercise (Figure 4). However, these incremental patterns were similar among the three trials, and there were no significant time × trial interactions for any of the markers of neutrophil activation.

### 3.7. Indirect Markers of Muscle Damage

There were significant main effects of time on serum myoglobin concentration and serum enzymatic activity of creatine kinase (Table 2). However, there were no significant time × trial interactions for these variables.

## 4. Discussion

In the present study, we demonstrated that CHO ingestion after endurance exercise did not enhance the exercise-induced increase in the neutrophil count, circulating cytokine levels, and markers of neutrophil activation and muscle damage, regardless of the amount of ingested CHO. Although these variables were not different among the three trials, plasma glucose and serum insulin concentrations during the recovery from exercise were higher in the HCHO trials than in the LCHO and PLA trials. Because CHO ingestion first 2 h after exercise enhances the rate of glycogen storage [25], and enhanced glycogen synthesis after exercise lasts several hours in the presence of CHO availability and high insulin levels [30], the results of the present study suggest that the ingestion of a high amount of CHO first 2 h after endurance exercise provides a favorable condition for recovery from endurance exercise without an increase in inflammatory responses and markers of muscle damage.

Although CHO ingestion before/during prolonged endurance exercise attenuates the exercise-induced increase in neutrophil count [11,12,13,14,15], the post-exercise ingestion of CHO did not influence the neutrophil count in the present study. During moderate- to high-intensity (60–85% $\overset{\cdot }{\text{V}}$O_2_ max) endurance exercise, stress hormones, such as cortisol and growth hormone [2,12,13,31], as well as IL-6 [2,11,14,16,17,18,31], which stimulate lipolysis and fat oxidation [32], increase to enhance fatty acid availability and maintain the plasma glucose levels [32,33]. Because cortisol and IL-6 promote neutrophil mobilization from the bone marrow [34,35], the neutrophil count increases 1–3 h after the end of endurance exercise [2,8,13]. Therefore, CHO ingestion before/during exercise might attenuate the exercise-induced increase in neutrophil count by suppressing cortisol and IL-6 responses, as shown in several studies [12,13,15]. Conversely, Costa et al. reported that CHO ingestion after endurance exercise did not influence the plasma cortisol concentrations during the recovery phase [26,27]. We also found that CHO ingestion after endurance exercise did not alter the exercise-induced IL-6 response (Figure 2). Therefore, neutrophil count might not have been influenced by CHO ingestion after endurance exercise because cortisol and IL-6 concentrations were not affected by post-exercise CHO ingestion.

Similarly, there were no differences in plasma concentrations of chemotactic factors, such as MCP-1, IL-8, and C5a among the three trials, suggesting that CHO ingestion after endurance exercise does not promote the migration and infiltration of neutrophils and monocytes to skeletal muscle. Furthermore, because the markers of muscle damage were not different among the three trials, CHO ingestion after endurance exercise also may not promote inflammation and muscle damage. Several studies have investigated the effect of CHO ingestion on exercise-induced chemokine production [11,12,14,17,21,36], of which three studies reported that CHO ingestion modulated exercise-induced chemokine production. Nieman et al. demonstrated the inhibitory effect of CHO ingestion on the expression of IL-8 mRNA in skeletal muscle after 3 h of running [11]; however, they also reported that CHO ingestion before/during marathon race enhanced exercise-induced increase in plasma IL-8 concentration [12]. Conversely, Ross et al. demonstrated that CHO ingestion after eccentric exercise enhanced the expression of MCP-1 and IL-8 mRNA in skeletal muscle [21]. These conflicting findings suggest the complex interaction between exercise and CHO ingestion in chemokine production. The difference in experimental protocols, such as exercise mode and intensity, the timing and the amount of CHO ingestion, and participant characteristics might influence the variable chemokine responses to exercise with CHO ingestion.

Plasma concentrations of the markers of neutrophil activation such as elastase, myeloperoxidase, and calprotectin were not different among the three trials. Several studies demonstrated that CHO ingestion modulated exercise-induced neutrophil activation that was evaluated by ex vivo experiments [13,19,26,27,37]. Conversely, plasma concentrations of myeloperoxidase, elastase, and calprotectin that are released from neutrophils and monocytes in response to a variety of inflammatory conditions [38], reflect neutrophil and monocyte activation in vivo. Because previous studies have reported conflicting findings on the effect of CHO ingestion before/during exercise on these markers of neutrophil activation [14,17,36], further studies are needed to elucidate whether CHO ingestion influences exercise-induced neutrophil activation in vivo.

The present study has several limitations. First, because only plasma samples were available, we did not measure neutrophil count and concentrations of cytokines and markers of neutrophil activation in skeletal muscle tissue [6,7,24]. There is a possibility that CHO ingestion after exercise only influences local tissue inflammation, but not systemic inflammation. Second, because we used a crossover design, the order of the three trials may have influenced the exercise-induced inflammatory responses and muscle damage. Actually, adaptation to a bout of ergometer cycling exercise was observed; neutrophil counts in the first trial were larger than those in the second and third trials, although there was no significant time × order interaction (*p* = 0.156). Although the order of the three trials was randomized in the present study, parallel design trials should be conducted in the future. Third, we only demonstrated that the effects of CHO ingestion after moderate-intensity endurance exercise on inflammatory responses in participants with moderate fitness levels, and our findings might not apply to high-intensity endurance exercise that is involving athletes with high fitness levels. Furthermore, although exercise-induced inflammatory responses and muscle damage differ by exercise mode (e.g., running vs. cycling, continuous vs. intermittent), we examined the effect of CHO ingestion on the exercise-induced inflammatory responses and muscle damage, only after continuous ergometer cycling. Actually, indirect markers of muscle damage were largely unchanged by 60 min of cycling exercise at 70% VO2 max, whereas high-intensity or prolonged running strikingly increased the levels of these markers [18,39]. Further investigations are needed to verify that our findings can be generalized, regardless of intensity and the mode of endurance exercise and the fitness levels of participants.

## 5. Conclusions

In conclusion, we revealed that CHO ingestion after endurance exercise did not enhance exercise-induced increase in neutrophil count, circulating cytokine levels, and markers of neutrophil activation and muscle damage, regardless of the amount of CHO that was ingested. Furthermore, the ingestion of a high amount of CHO stimulated insulin secretion and maintained high plasma glucose concentrations during the recovery phase when compared to the ingestion of a low amount of CHO. These findings suggest that the ingestion of a high amount of CHO after endurance exercise provides a favorable condition for the recovery from endurance exercise without an increase in inflammatory responses and markers of muscle damage.

## Figures and Tables

**Table 1:** Changes in circulating blood cell counts in response to endurance exercise.

Variables	Trial	Pre	Post	0.5 h	1 h	2 h	Analysis of Variance (ANOVA) *p*-Values		
							Time	Trial	Interaction
			†		†	†			
Total leukocytes (×10^9^ cells/L)	HCHO	5.6 (1.4)	6.8 (2.0)	7.5 (3.1)	8.5 (2.8)	8.7 (2.2)	0.001	0.608	0.685
	LCHO	5.6 (1.8)	7.0 (1.8)	7.6 (2.9)	8.9 (3.0)	8.8 (2.3)			
	PLA	5.6 (1.3)	7.6 (1.3)	8.0 (3.1)	8.7 (2.9)	9.1 (2.2)			
			†						
Lymphocytes (×10^9^ cells/L)	HCHO	2.0 (0.6)	2.5 (0.8)	1.7 (0.5)	1.6 (0.4)	1.7 (0.4)	<0.001	0.953	0.656
	LCHO	1.9 (0.7)	2.7 (0.5)	1.7 (0.4)	1.7 (0.4)	1.8 (0.4)			
	PLA	1.8 (0.5)	2.6 (0.8)	1.8 (0.6)	1.7 (0.4)	1.7 (0.4)			
Monocytes (×10^9^ cells/L)	HCHO	0.7 (0.3)	0.8 (0.4)	0.7 (0.3)	0.7 (0.2)	0.7 (0.1)	0.718	0.525	0.940
	LCHO	0.7 (0.3)	0.8 (0.4)	0.7 (0.2)	0.7 (0.3)	0.7 (0.2)			
	PLA	0.7 (0.3)	0.8 (0.3)	0.8 (0.3)	0.8 (0.3)	0.7 (0.2)			
			†		†	†			
Neutrophils (×10^9^ cells/L)	HCHO	2.8 (0.9)	3.5 (1.1)	5.1 (2.9)	6.2 (2.8)	6.3 (2.2)	0.001	0.691	0.624
	LCHO	2.9 (1.0)	3.5 (1.3)	5.3 (2.6)	6.5 (2.7)	6.3 (2.3)			
	PLA	3.1 (0.8)	4.2 (1.2)	5.4 (2.9)	6.3 (2.7)	6.7 (2.2)			
					†	†			
Neutrophil/lymphocyte ratio	HCHO	1.5 (0.5)	1.5 (0.5)	3.0 (1.6)	3.9 (1.6)	3.8 (1.6)	<0.001	0.840	0.784
	LCHO	1.7 (0.6)	1.3 (0.5)	3.2 (1.3)	3.9 (1.4)	3.8 (1.5)			
	PLA	1.9 (1.1)	1.8 (0.9)	3.2 (1.7)	3.8 (1.5)	4.1 (1.5)			

Data are presented as mean (standard deviation). HCHO, high-carbohydrate trial; LCHO, low-carbohydrate trial; PLA, placebo trial; Pre, pre-exercise; Post, immediately after exercise; 0.5 h, 0.5 h after exercise; 1 h, 1 h after exercise; 2 h, 2 h after exercise. † *p* < 0.05 vs. Pre.

**Table 2:** Changes in serum concentration and enzymatic activity of indirect markers of muscle damage in response to endurance exercise.

Variables	Trial	Pre	Post	0.5 h	1 h	2 h	ANOVA *p*-Values		
							Time	Trial	Interaction
				†	†	†			
Creatine kinase (IU/L)	HCHO	181 (79)	188 (80)	198 (91)	195 (86)	192 (83)	<0.001	0.198	0.638
	LCHO	185 (137)	184 (128)	206 (155)	193 (136)	195 (122)			
	PLA	175 (45)	181 (43)	190 (49)	188 (50)	186 (47)			
Myoglobin (ng/mL)	HCHO	41 (5)	40 (7)	47 (14)	47 (16)	47 (24)	0.031	0.033	0.471
	LCHO	36 (8)	30 (6)	51 (32)	60 (57)	78 (112)			
	PLA	39 (8)	36 (10)	45 (12)	54 (31)	60 (46)			

Data are presented as mean (standard deviation). HCHO, high-carbohydrate trial; LCHO, low-carbohydrate trial; PLA, placebo trial; Pre, pre-exercise; Post, immediately after exercise; 0.5 h, 0.5 h after exercise; 1 h, 1 h after exercise; 2 h, 2 h after exercise. † *p* < 0.05 vs. Pre.

## References

[1] Peake J., Nosaka K., Suzuki K. (2005). Characterization of inflammatory responses to eccentric exercise in humans. Exerc. Immunol. Rev..

[2] Suzuki K., Totsuka M., Nakaji S., Yamada M., Kudoh S., Liu Q., Sugawara K., Yamaya K., Sato K. (1999). Endurance exercise causes interaction among stress hormones, cytokines, neutrophil dynamics, and muscle damage. J. Appl. Physiol..

[3] Kawanishi N., Mizokami T., Niihara H., Yada K., Suzuki K. (2016). Neutrophil depletion attenuates muscle injury after exhaustive exercise. Med. Sci. Sports Exerc..

[4] Kawanishi N., Mizokami T., Niihara H., Yada K., Suzuki K. (2016). Macrophage depletion by clodronate liposome attenuates muscle injury and inflammation following exhaustive exercise. Biochem. Biophys. Rep..

[5] Pillon N.J., Bilan P.J., Fink L.N., Klip A. (2013). Cross-talk between skeletal muscle and immune cells: Muscle-derived mediators and metabolic implications. Am. J. Physiol. Endocrinol. Metab..

[6] Mittal M., Siddiqui M.R., Tran K., Reddy S.P., Malik A.B. (2014). Reactive oxygen species in inflammation and tissue injury. Antioxid. Redox Signal..

[7] Wright H.L., Moots R.J., Bucknall R.C., Edwards S.W. (2010). Neutrophil function in inflammation and inflammatory diseases. Rheumatology (Oxford).

[8] Suzuki K., Sato H., Kikuchi T., Abe T., Nakaji S., Sugawara K., Totsuka M., Sato K., Yamaya K. (1996). Capacity of circulating neutrophils to produce reactive oxygen species after exhaustive exercise. J. Appl. Physiol..

[9] Tidball J.G. (2005). Inflammatory processes in muscle injury and repair. Am. J. Physiol. Regul. Integr. Comp. Physiol..

[10] Peake J., Suzuki K., Wilson G., Hordern M., Nosaka K., Mackinnon L., Coombes J.S. (2005). Exercise-induced muscle damage, plasma cytokines, and markers of neutrophil activation. Med. Sci. Sports Exerc..

[11] Nieman D.C., Davis J.M., Henson D.A., Walberg-Rankin J., Shute M., Dumke C.L., Utter A.C., Vinci D.M., Carson J.A., Brown A. (2003). Carbohydrate ingestion influences skeletal muscle cytokine mrna and plasma cytokine levels after a 3-h run. J. Appl. Physiol..

[12] Nieman D.C., Henson D.A., Smith L.L., Utter A.C., Vinci D.M., Davis J.M., Kaminsky D.E., Shute M. (2001). Cytokine changes after a marathon race. J. Appl. Physiol..

[13] Nieman D.C., Nehlsen-Cannarella S.L., Fagoaga O.R., Henson D.A., Utter A., Davis J.M., Williams F., Butterworth D.E. (1998). Effects of mode and carbohydrate on the granulocyte and monocyte response to intensive, prolonged exercise. J. Appl. Physiol..

[14] Peake J., Peiffer J.J., Abbiss C.R., Nosaka K., Laursen P.B., Suzuki K. (2008). Carbohydrate gel ingestion and immunoendocrine responses to cycling in temperate and hot conditions. Int. J. Sport Nutr. Exerc. Metab..

[15] Peake J., Wilson G., Mackinnon L., Coombes J.S. (2005). Carbohydrate supplementation and alterations in neutrophils, and plasma cortisol and myoglobin concentration after intense exercise. Eur. J. Appl. Physiol..

[16] Nehlsen-Cannarella S.L., Fagoaga O.R., Nieman D.C., Henson D.A., Butterworth D.E., Schmitt R.L., Bailey E.M., Warren B.J., Utter A., Davis J.M. (1997). Carbohydrate and the cytokine response to 2.5 h of running. J. Appl. Physiol..

[17] Suzuki K., Hashimoto H., Oh T., Ishijima T., Mitsuda H., Peake J.M., Sakamoto S., Muraoka I., Higuchi M. (2013). The effects of sports drink osmolality on fluid intake and immunoendocrine responses to cycling in hot conditions. J. Nutr. Sci. Vitaminol. (Tokyo).

[18] Suzuki K., Nakaji S., Yamada M., Liu Q., Kurakake S., Okamura N., Kumae T., Umeda T., Sugawara K. (2003). Impact of a competitive marathon race on systemic cytokine and neutrophil responses. Med. Sci. Sports Exerc..

[19] Scharhag J., Meyer T., Gabriel H.H., Auracher M., Kindermann W. (2002). Mobilization and oxidative burst of neutrophils are influenced by carbohydrate supplementation during prolonged cycling in humans. Eur. J. Appl. Physiol..

[20] Valentine R.J., Saunders M.J., Todd M.K., St Laurent T.G. (2008). Influence of carbohydrate-protein beverage on cycling endurance and indices of muscle disruption. Int. J. Sport Nutr. Exerc. Metab..

[21] Ross M.L., Halson S.L., Suzuki K., Garnham A., Hawley J.A., Cameron-Smith D., Peake J.M. (2010). Cytokine responses to carbohydrate ingestion during recovery from exercise-induced muscle injury. J. Interferon Cytokine Res..

[22] Afroundeh R., Siahkouhian M., Khalili A. (2010). The effect of post-exercise carbohydrate ingestion on inflammatory responses to short time, high-force eccentric exercise. J. Sports Med. Phys. Fit..

[23] Depner C.M., Kirwan R.D., Frederickson S.J., Miles M.P. (2010). Enhanced inflammation with high carbohydrate intake during recovery from eccentric exercise. Eur. J. Appl. Physiol..

[24] Burke L.M., Kiens B., Ivy J.L. (2004). Carbohydrates and fat for training and recovery. J. Sports Sci..

[25] Ivy J.L., Katz A.L., Cutler C.L., Sherman W.M., Coyle E.F. (1988). Muscle glycogen synthesis after exercise: Effect of time of carbohydrate ingestion. J. Appl. Physiol..

[26] Costa J., Walters R., Bilzon J.L., Walsh N.P. (2011). Effects of immediate postexercise carbohydrate ingestion with and without protein on neutrophil degranulation. Int. J. Sport Nutr. Exerc. Metab..

[27] Costa J., Oliver S.J., Laing S.J., Waiters R., Bilzon J.L., Walsh N.P. (2009). Influence of timing of postexercise carbohydrate-protein ingestion on selected immune indices. Int. J. Sport Nutr. Exerc. Metab..

[28] Dill D.B., Costill D.L. (1974). Calculation of percentage changes in volumes of blood, plasma, and red cells in dehydration. J. Appl. Physiol..

[29] Faul F., Erdfelder E., Lang A.G., Buchner A. (2007). G*power 3: A flexible statistical power analysis program for the social, behavioral, and biomedical sciences. Behav. Res. Methods.

[30] Ivy J.L. (1991). Muscle glycogen synthesis before and after exercise. Sports Med..

[31] Suzuki K., Yamada M., Kurakake S., Okamura N., Yamaya K., Liu Q., Kudoh S., Kowatari K., Nakaji S., Sugawara K. (2000). Circulating cytokines and hormones with immunosuppressive but neutrophil-priming potentials rise after endurance exercise in humans. Eur. J. Appl. Physiol..

[32] Van Hall G., Steensberg A., Sacchetti M., Fischer C., Keller C., Schjerling P., Hiscock N., Moller K., Saltin B., Febbraio M.A. (2003). Interleukin-6 stimulates lipolysis and fat oxidation in humans. J. Clin. Endocrinol. Metab..

[33] Petersen E.W., Carey A.L., Sacchetti M., Steinberg G.R., Macaulay S.L., Febbraio M.A., Pedersen B.K. (2005). Acute IL-6 treatment increases fatty acid turnover in elderly humans in vivo and in tissue culture in vitro. Am. J. Physiol. Endocrinol. Metab..

[34] Cavalcanti D.M., Lotufo C.M., Borelli P., Ferreira Z.S., Markus R.P., Farsky S.H. (2007). Endogenous glucocorticoids control neutrophil mobilization from bone marrow to blood and tissues in non-inflammatory conditions. Br. J. Pharmacol..

[35] Liu F., Poursine-Laurent J., Wu H.Y., Link D.C. (1997). Interleukin-6 and the granulocyte colony-stimulating factor receptor are major independent regulators of granulopoiesis in vivo but are not required for lineage commitment or terminal differentiation. Blood.

[36] Hashimoto H., Ishijima T., Hayashida H., Suzuki K., Higuchi M. (2014). Menstrual cycle phase and carbohydrate ingestion alter immune response following endurance exercise and high intensity time trial performance test under hot conditions. J. Int. Soc. Sports Nutr..

[37] Bishop N.C., Walsh N., Scanlon G.A. (2003). Effect of prolonged exercise and carbohydrate on total neutrophil elastase content. Med. Sci. Sports Exerc..

[38] Yui S., Nakatani Y., Mikami M. (2003). Calprotectin (s100a8/s100a9), an inflammatory protein complex from neutrophils with a broad apoptosis-inducing activity. Biol. Pharm. Bull..

[39] Peake J., Wilson G., Hordern M., Suzuki K., Yamaya K., Nosaka K., Mackinnon L., Coombes J.S. (2004). Changes in neutrophil surface receptor expression, degranulation, and respiratory burst activity after moderate- and high-intensity exercise. J. Appl. Physiol..

